# Circulating cell‐free circRNA panel predicted tumorigenesis and development of colorectal cancer

**DOI:** 10.1002/jcla.24431

**Published:** 2022-04-14

**Authors:** Le Qi, Ying Pan, Min Tang, Xi Chen

**Affiliations:** ^1^ Department of Gastroenterology The First Affiliated Hospital of Anhui Medical University Hefei China; ^2^ Department of Gastroenterology The Fourth Affiliated Hospital of Anhui Medical University Hefei China; ^3^ Department of Gastroenterology The Fourth Affiliated Hospital of Nanjing Medical University Nanjing China

**Keywords:** cell‐free, circRNA, colorectal adenoma, fingerprint, plasma

## Abstract

**Background:**

Colorectal cancer (CRC) is reported with high morbidity and mortality. Currently, the sensitivity of diagnostic markers for colorectal cancer is low. Therefore, further exploration of new plasma diagnostic markers for early detection of colorectal cancer is of great value. We aimed to explore potential circRNAs in plasma as biomarkers for early diagnosis of CRC.

**Methods:**

We employed the circRNA microarray to investigate dysregulated circRNAs in plasma samples of CRC patients, colorectal adenoma patients (CRA), and healthy controls. Through in‐depth analysis, significantly differentially expressed circRNAs were screened as candidate targets.

**Results:**

Eight circRNAs (hsa_circ_104885, hsa_circ_100185, hsa_circ_103171, hsa_circ_001978, hsa_circ_105039, hsa_circ_103627, hsa_circ_101717, and hsa_circ_104192) were obtained as candidate circRNAs with upregulation in CRC comparing with both CRA and healthy control. Through detecting the plasma expression levels of eight candidate targets, we identified three circRNA (hsa_circ_001978, hsa_circ_105039, and hsa_circ_103627) with increased level which were consistent with the microarray results in training set. Further validation found the circRNA panel was consistent with training set. The ROC curve also revealed a high diagnostic ability of hsa_circ_001978, hsa_circ_105039, and hsa_circ_103627 in predicted the CRC from CRA patients (AUC = 0.966) as well as healthy controls (AUC = 0.969).

**Conclusion:**

Our data suggest that hsa_circ_001978, hsa_circ_105039, and hsa_circ_103627 might be a CRC‐specific biomarker for early diagnosis.

## Graphical abstract

(1) Three circRNA panel predicted CRC from CRA and healthy control. (2) Three circRNA panel could be a CRC‐specific marker with diagnostic value and provide insights into targeted therapy in the treatment of CRC.

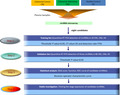

## 1 INTRODUCTION

Colorectal cancer (CRC) is one of the most common gastrointestinal malignancies, ranking third in males and second in females.^1^, ^2^ CRC is characterized by high morbidity and mortality.^3^ As reported, nearly about 1.8 million new diagnosed cases of colorectal cancer and about 880,000 deaths each year worldwide.^4^ According to statistics, the incidence and mortality rate of colorectal cancer in developed countries has been ranked in the third and second malignant tumors. In China, with economic development, the incidence of colorectal cancer is increasing year by year.^5^ Most patients have been diagnosed with local advanced or distant metastasis, especially liver metastasis, with poor treatment effect and poor prognosis.^2^ In recent decades, researchers have developed many effective methods for the diagnosis and treatment of colorectal cancer, which significantly improve the survival of colorectal cancer patients. However, the overall prognosis of colorectal cancer patients is still poor.^6^ Due to the lack of specific clinical manifestations, simple, and fast diagnostic methods and accurate evaluation methods, diagnosis, and treatment of colorectal cancer is still difficult. There is a long way to go in the diagnosis and treatment of colorectal cancer in China. Therefore, to improve the prognosis of patients with colorectal cancer, the key point is to improve the efficiency of early diagnosis of colorectal cancer. Developing effective minimally invasive diagnostic markers to detect colorectal cancer at an earlier stage, patients with colorectal cancer can receive timely treatment, effectively improve the survival of patients, which has important clinical research value.

Recently discovered circRNAs consist mainly of exon‐derived transcripts produced by non‐collinear reverse splicing.^7^ Based on whether they can be translated, circRNAs can be divided into non‐coding circRNAs and coding circRNAs.^7^, ^8^, ^9^ The circRNA is widely expressed in a variety of human cells and plays a key role in the regulation of post‐transcriptional gene expression.^10^ Different from linear RNA, circRNA harbored a closed‐loop structure, stable expression, is not easy to degrade, and is affected by RNA exonucleases.^11^ The circRNA has miRNA binding sites and acts as a miRNA sponge, blocking or reducing miRNA expression and promoting the expression of target mRNA.^12^ There is an increasing evidence that circRNA can be used as biomarkers for early diagnosis and prognostic prediction in various diseases.^13^ Abnormal circRNA expression in peripheral blood can be used as a biomarker of rectal cancer.^14^ Similarly, circRNA can also be used as an early diagnostic marker for some non‐neoplastic diseases, such as Parkinson's disease, and can be used to monitor the severity of the disease.^15^ Besides, recent colorectal cancer tumor markers, such as CEA and CA19‐9, have been confirmed to have obvious limitations in their diagnostic sensitivity and specificity, especially in patients with early colorectal cancer, whose diagnostic efficacy is weak. However, it can predict postoperative metastasis and recurrence of colorectal cancer.^16^ Based on this, it is necessary to explore novel biomarker for the early diagnosis of colorectal cancer.

In this project, we anchored circRNA as the research object and combined with the characteristics of stable expression of circRNA, we believe that circRNA might be used as a potential biomarker. We screened the differentially expressed circRNA in the plasma of colorectal cancer patients by high‐throughput detection and compared them with CRA and healthy people, respectively. The diagnostic efficacy of circRNA with clear differentially expressed circRNA was tested by risk score analysis.

## 2 MATERIALS AND METHODS

### 2.1 Clinical samples

We selected patients diagnosed with colorectal cancer in the First/Fourth Affiliated Hospital of Anhui Medical University and the Fourth Affiliated Hospital of Nanjing Medical University from September 2019 to October 2021 and collected plasma samples from 100 patients before endoscopic treatment. In addition, we also selected plasma samples from 100 patients diagnosed with colorectal adenoma during the same period and matched plasma samples from 100 healthy people who underwent endoscopic physical examination. All the participants signed informed consent and agreed to obtain basic clinical information of the patients when taking blood samples. The peripheral blood samples of patients were collected in the before operation. All studies in this project have been approved by the Ethics Committee of Anhui Medical University and carried out in strict accordance with the Ethics Committee and the Helsinki Declaration.

### 2.2 Study design

The circRNA microarray was employed by using Arraystar Circular RNA Microarray Version 2.0. The plasma samples were obtained from four CRC patients, four patients with CRA and three healthy controls were conducted to investigate elevated circRNAs in the plasma of CRC patients by comparing with both CRA patients and healthy control group.

Next, we conducted an in‐depth analysis of the differentially expressed circRNA detected by microarray. Intersection analysis was conducted for circRNA with high differential expression compared with CRA group and circRNA with elevated expression compared with healthy people, and the final differential circRNA was obtained as a candidate. Subsequently, plasma samples from 20 CRC patients and 20 healthy controls were randomly selected for preliminary validation to obtain circRNA with differential expression in a small sample. Based on the above results, a large sample (80 cases in each group) was used for revalidation. Finally, the sensitivity and specificity of candidate patients and their combinations in CRC diagnosis were calculated by setting training set and validation set. The receiver operating characteristic (ROC) curve analysis was applied.

### 2.3 RNA isolation and qRT‐PCR

All blood samples were stored by EDTA anticoagulant tube, and plasma samples were obtained by 1000 *g* centrifugation for 10 min within half an hour after blood samples were taken. All plasma samples were stored in the −80°C refrigerator. Total RNA was extracted by Trizol method, and circRNA was enriched by RNase treatment after RNA quality control detection. The RNase‐treated RNA were retrotranscribed using random primers method, and the reverse transcribed cDNA was amplified using a SYBR^®^Premix Ex Taq™ kit (Takara). Finally, the amplification curve was compared with the internal reference to obtain the relative expression level, and triplicates were used to detect each samples. ABI7900 (ABI) was used for amplification.

### 2.4 Statistical analysis

In this study, continuous variables were analyzed using the student *t* test, and categorical variables were analyzed using the chi‐square test. All the experiment assay was repeated three times in triplicate. We have also tested the homoscedasticity in all the data obtained. After confirmed the homoscedasticity, after that, a parametric test (*t* test, ANOVA) was applied. In analyzing the sensitivity and specificity of diagnostic markers, we quantified the area under the receiver operating characteristic (ROC) curve. Specific methods of risk stratification analysis are described in previous reports. STATA14.0 was used for statistical analysis, and GraphPad Prism 8.0 was used for graph presentation. In all statistical analyses, *p* < 0.05 was considered statistically significant.

## 3 RESULTS

### 3.1 High‐throughput microarray detection of plasma circRNAs

Totally, 100 patients diagnosed with CRC were enrolled with 100 paired healthy controls. 100 patients diagnosed with colorectal adenoma was also participated. The detailed clinical information is listed in Table 1. Based on clinical information analysis, it was found that there were no significant statistical differences in age and gender among the groups in this study. We further presented subgroup information based on clinical characteristics of tumor patients, including tumor size, tumor differentiation degree, and tumor TNM analysis.

**TABLE 1:** Clinical characteristics of CRC, CRA patients and healthy controls

Characteristics	CRC	CRA	Control	*p* Value
*N*	100	100	100	
Age Mean (SE) year	41.78(0.36)	41.89(0.43)	42.11(0.22)	0.384^a^
Sex (male/female)	78/22	69/31	72/28	0.345^b^
Differentiation grade				
Well	17			
Moderate	78			
Poorly	5			
Tumor Size(cm)				
≤3 cm	76			
>3 cm	24			
Metastasis				
Yes	38			
No	62			
TNM stage				
Stage I, II	29			
Stage III, IV	71			

^a^ Student *t* test.

^b^ Chi‐square test.

**FIGURE 1:**
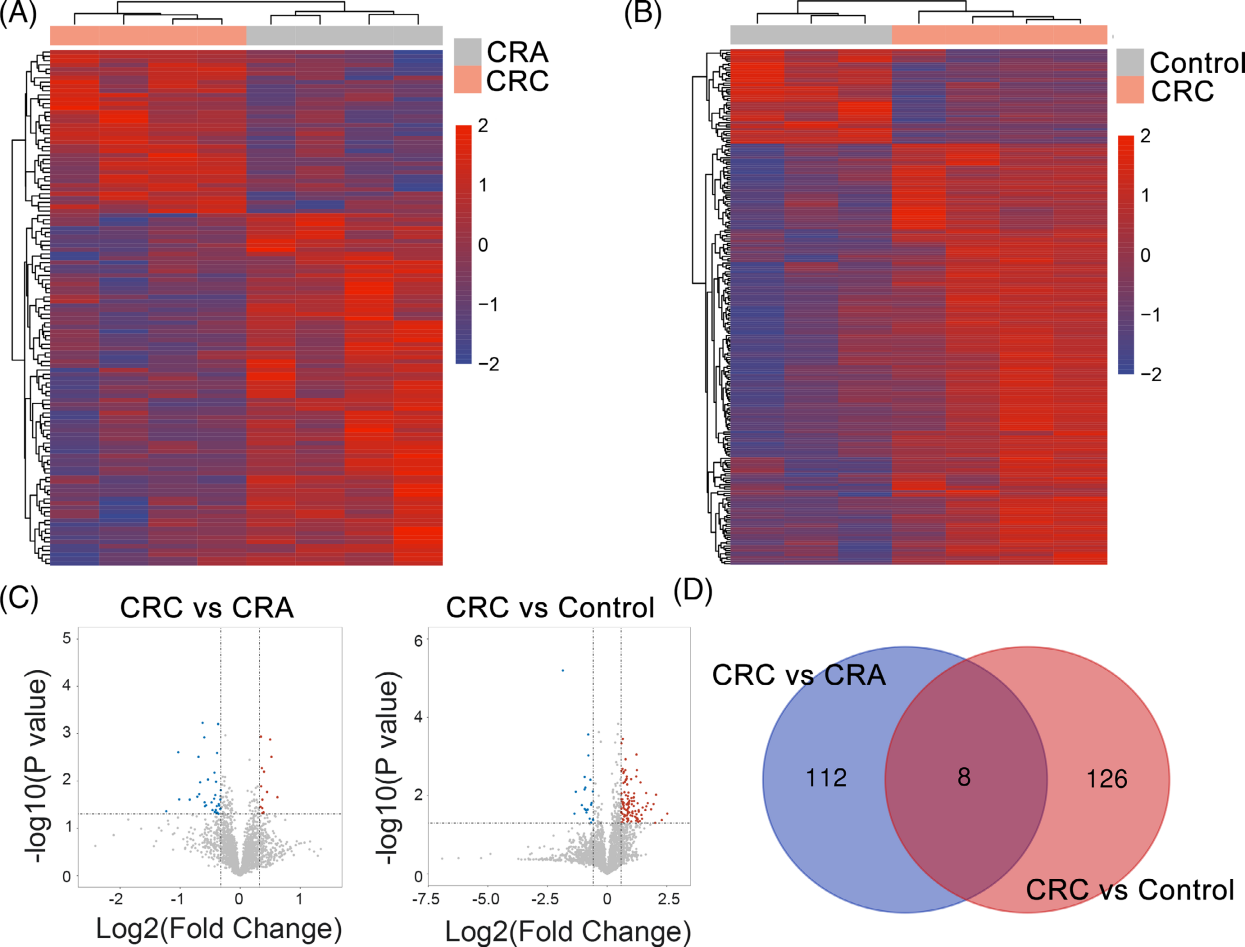
Circulating circRNAs expression landscape of in CRC, CRA, and healthy control samples. (A and B) Cluster analysis of differentially expressed circRNA in each group. (C) Volcano blot presented differentially expressed circRNA. (D) Intersection matching analysis of CRA group and healthy control compared with CRC group. CRA, colorectal adenomas; CRC, colorectal cancer

After obtained the dysregulated circRNA, the following parameters were used for difference analysis for all circRNA: (1) *p *< 0.05, (2) the absolute value of CT detection <35, and (3) 75% detectable in all the samples. After the above screening, we obtained 134 circRNA with high expression in CRC compared with the CRA group.120 circRNA were elevated in CRC compared with healthy subjects. Further, the enrichment of differentially expressed circRNA in each group was presented by clustering map, and the general distribution of differentially expressed circRNA was shown by volcanic map (Figure 1A–C). Further cross comparison was conducted for the above two groups of differentially expressed circRNAs, and 8 circRNAs with the highest expression in CRC were obtained (Figure 1D). We further selected the eight circRNAs as candidate biomarkers.

**FIGURE 2:**
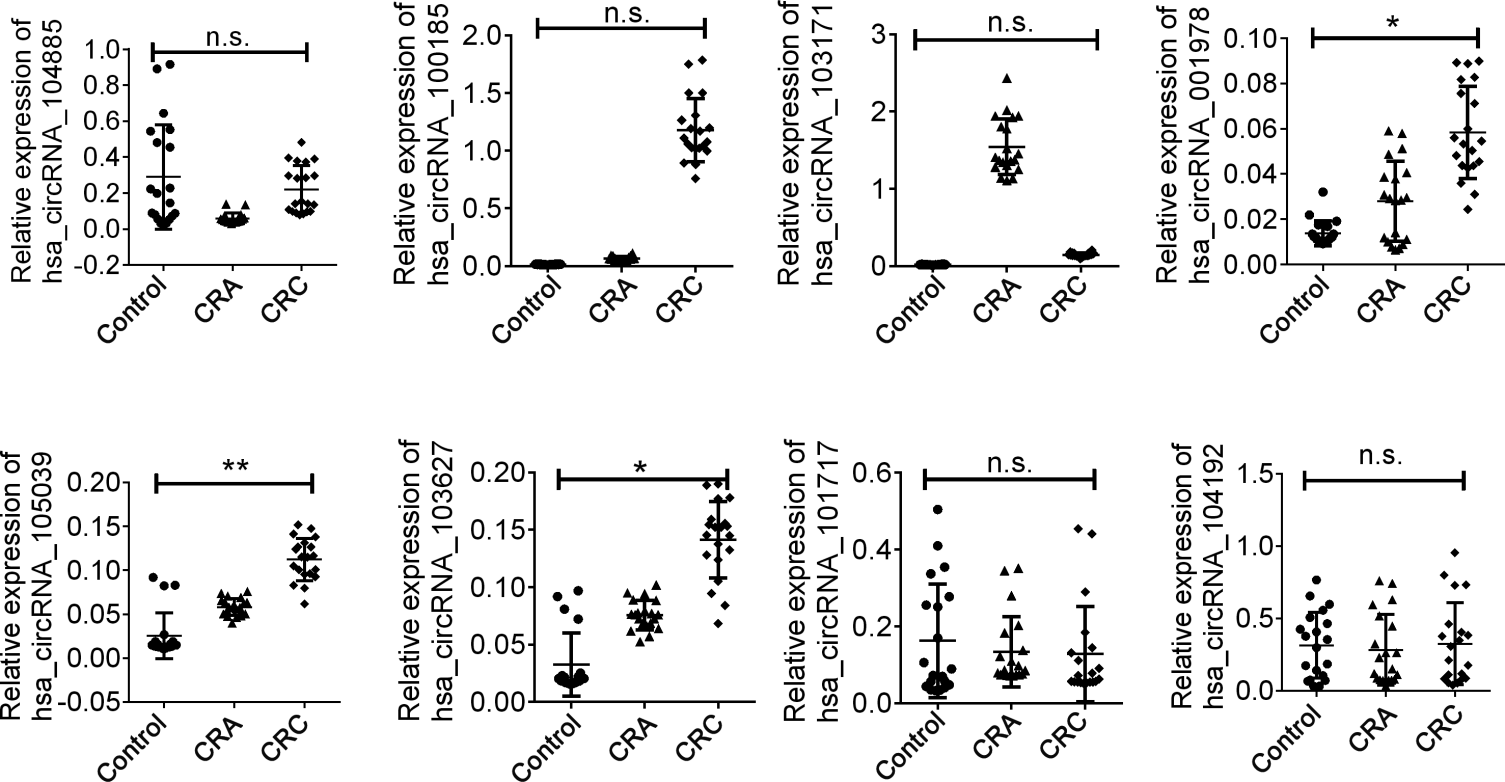
Relative expression of circRNA in training set. The eight circRNAs were examined in 20 paired samples of three group. Data was presented as mean ± SD, **indicated *p < *0.01. *indicated *p < *0.05, n.s, indicated no significance

We next employed an independent cohort to further validate 8 candidates. As presented in Figure 2, all the 8 circRNAs presented abundant amplification with the RT‐PCR assay. Among the eight candidates, three circRNAs including hsa_circ_104885, hsa_circ_101717, and hsa_circ_104192 presented no difference in any of the three group. Hsa_circ_100185 presented no difference between control group and CRA group while hsa_circ_103171 indicated no difference between control group and CRC group. The rest three circRNAs, including hsa_circ_001978, hsa_circ_105039, and hsa_circ_103627, were confirmed with significance and were employed for the following validation and might be effective markers for predicting CRC.

### 3.2 Risk score analysis for colorectal cancer biomarkers

**FIGURE 3:**
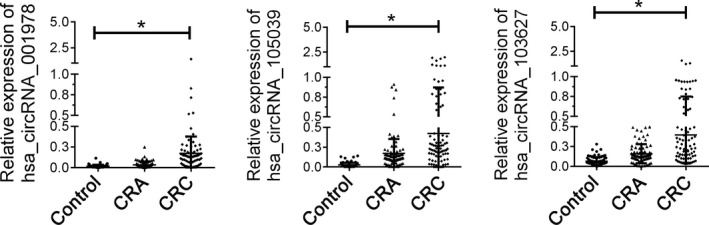
Relative expression of candidate circRNA in independent cohort. The eight circRNAs were examined in 80 paired plasma samples from healthy controls, CRA patients and CRC patients. **indicated *p < *0.01. *indicated *p < *0.05, n.s, indicated no significance

The panel of three circRNAs (hsa_circ_001978, hsa_circ_105039 and hsa_circ_103627) was further validated in an independent cohort including 80 samples in each group. Comparing with both CRA group and healthy control group, we found the three circRNA panel was evaluated in the plasma samples of CRC patients (Figure 3).

As mentioned above, based on the above study and multi‐step verification, we preliminarily found three circRNA panel might be potential biomarkers of CRC. We next employed the risk score analysis (RSA) to calculate the predicting ability for CRC and cancer‐free samples to evaluating the practical value of the panel of three circRNAs as CRC diagnostic markers. First, we calculated the risk value of each plasma sample through logistic regression model, whose risk score was taken as a parameter. Based on logistic regression analysis, we obtained the sum of sensitivity and specificity as the node of the maximum risk score, regarding as the cutoff. We classified the population as high‐risk group and low‐risk group. The high‐risk group was suggested to be colorectal cancer, and the low‐risk group was the control group (value = 4.31). We found the positive predicting value for CRC from healthy was 1.00 and the negative predicting value was 1.00. The same method was applied in validation set, and the obtained the positive predictive value and negative predictive value were 0.90 and 0.91, respectively, based on a cutoff value of 8.72 (Table 2).

**TABLE 2:** Risk score analysis of in CRC and cancer‐free control plasma samples

Score	0–4.31	4.31–8.72	PPV^a^	NPV^b^
Training set (*n =* 20)				
CRC	0	20	1.00	1.00
Control	20	0		
Validation set (*n =* 80)				
CRC	8	7	0.90	0.91
Control	72	73		

^a^ PPV, positive predictive value.

^b^ NPV, negative predictive value.

**FIGURE 4:**
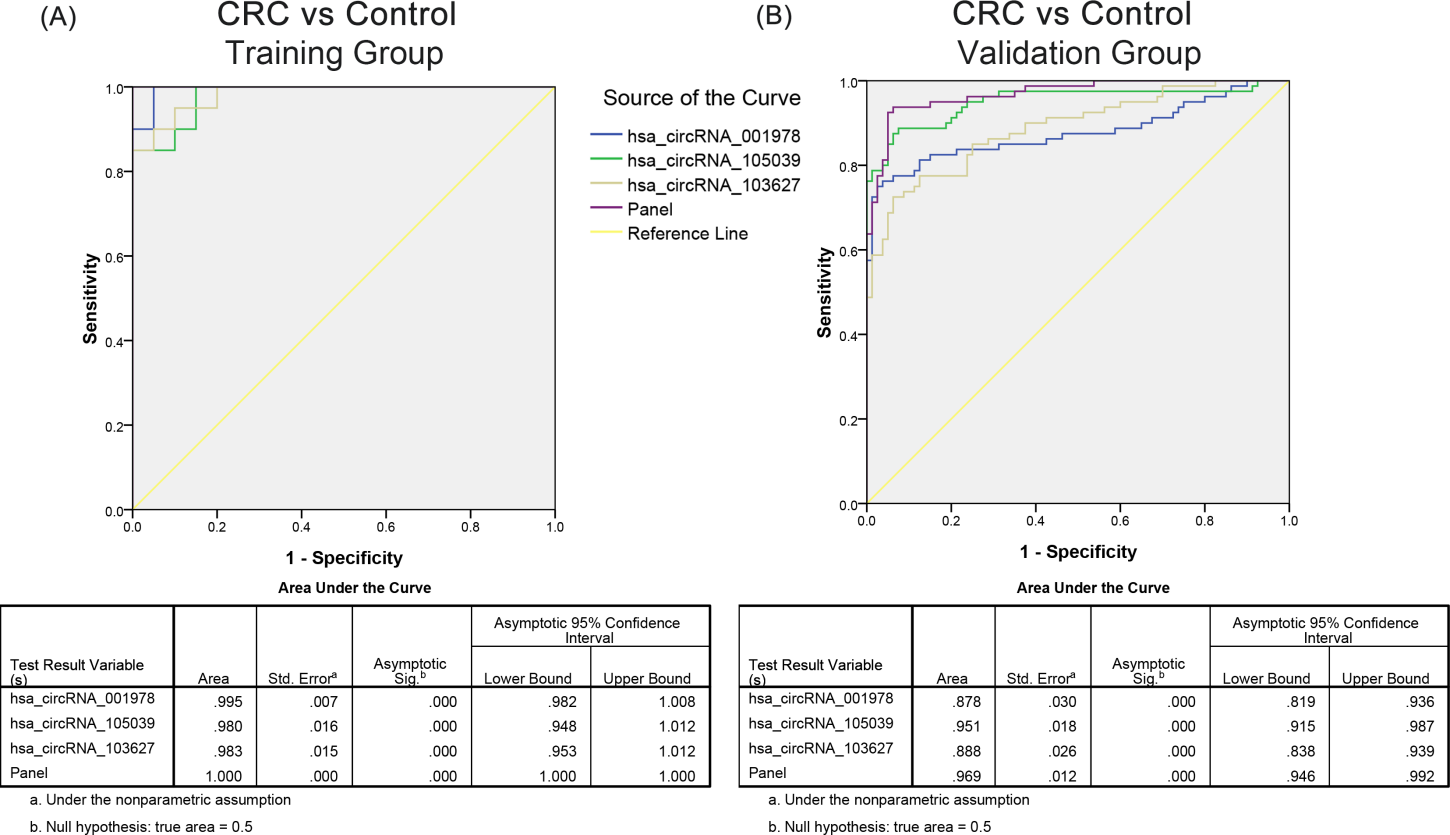
CircRNA panel predicted CRC from healthy controls. (A) Diagnostic efficacy of three circRNA panel as diagnostic marker for colorectal cancer in healthy population in training sets. (B) Diagnostic efficacy of three circRNA panel as biomarker of colorectal cancer in healthy population in validation set

The specific diagnostic efficacy of the above fingerprints was presented by ROC curves. As shown in Figure 4, the AUC of hsa_circ_001978, hsa_circ_105039, and hsa_circ_103627 and their combination was 0.995, 0.980, 0.983, and 1.000 in training set. When the sample size was expanded to 80 paired, the AUC for the non‐coding RNAs and their combination was 0.878, 0.951, 0.888 and 0.969.

**FIGURE 5:**
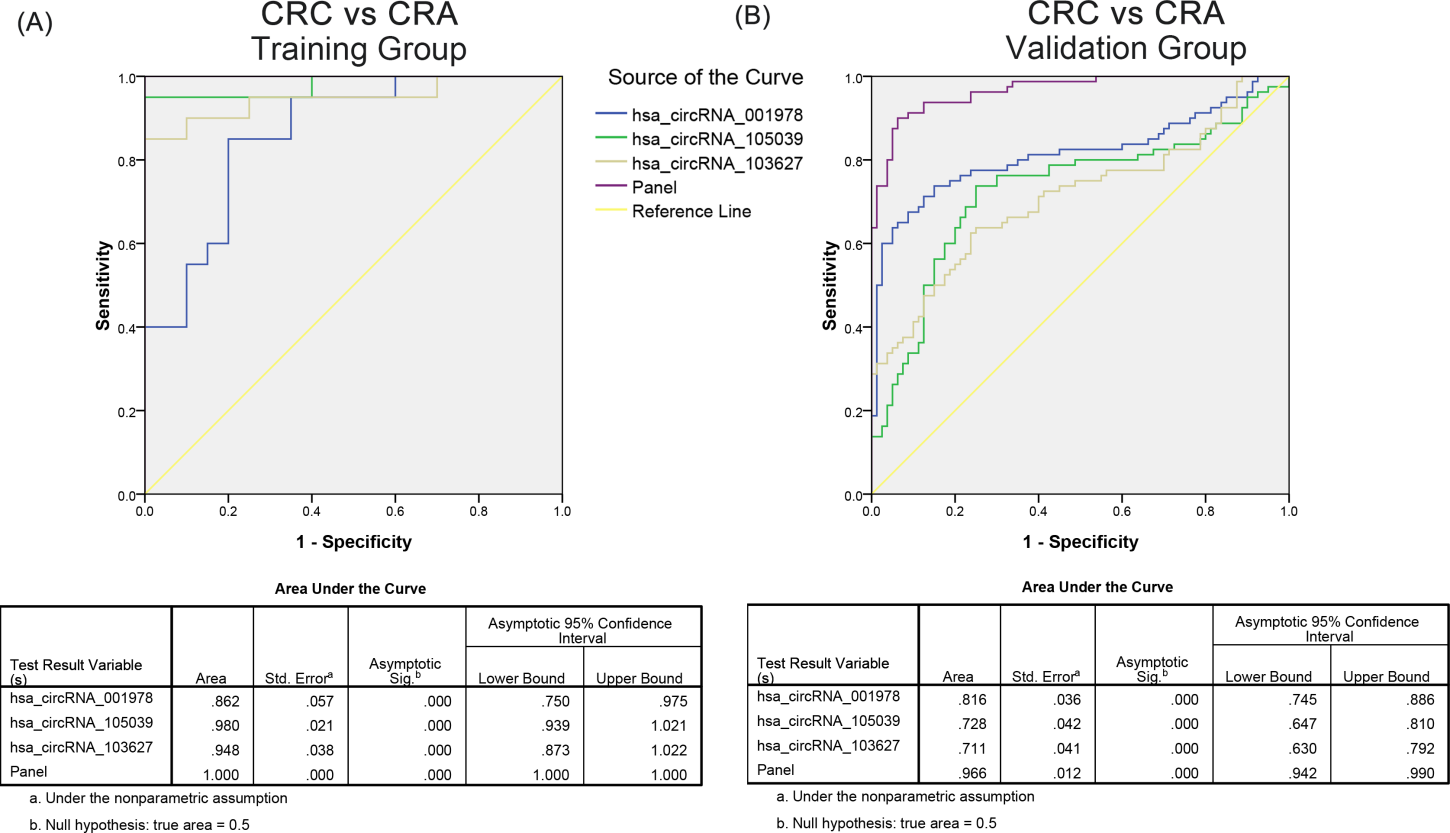
CircRNA panel predicted CRC from CRA. (A) Diagnostic efficacy of three circRNA panel as diagnostic marker for colorectal cancer in CRA patients in training sets. (B) Diagnostic efficacy of three circRNA panel as biomarker of colorectal cancer in CRA patients in validation set

The risk score analysis was also applied for analysis the predicting ability of the three circRNA in CRC from CRA. The positive predicting value (PPV) for CRC from healthy was 0.95, and the negative predicting value (NPV) was 0.90. Besides, the PPV and NPV in validation set was 0.86 and 0.85 (Table 3). The AUC of hsa_circ_001978, hsa_circ_105039, hsa_circ_103627 and their combination was 0.862, 0.980, 0.948, and 1.000 in training set, and was 0.816, 0.728, 0.711, and 0.966 in the 80 paired CRC samples, respectively (Figure 5).

**TABLE 3:** Risk score analysis of in CRC and CRA patients’ plasma samples

Score	0–5.02	5.02–10.55	PPV^a^	NPV^b^
Training set (*n =* 20)				
CRC	1	18	0.95	0.90
CRA	19	2		
Validation set (*n =* 80)				
CRC	11	68	0.86	0.85
CRA	69	12		

^a^ PPV, positive predictive value.

^b^ NPV, negative predictive value.

### 3.3 Expression stability validation

**FIGURE 6:**
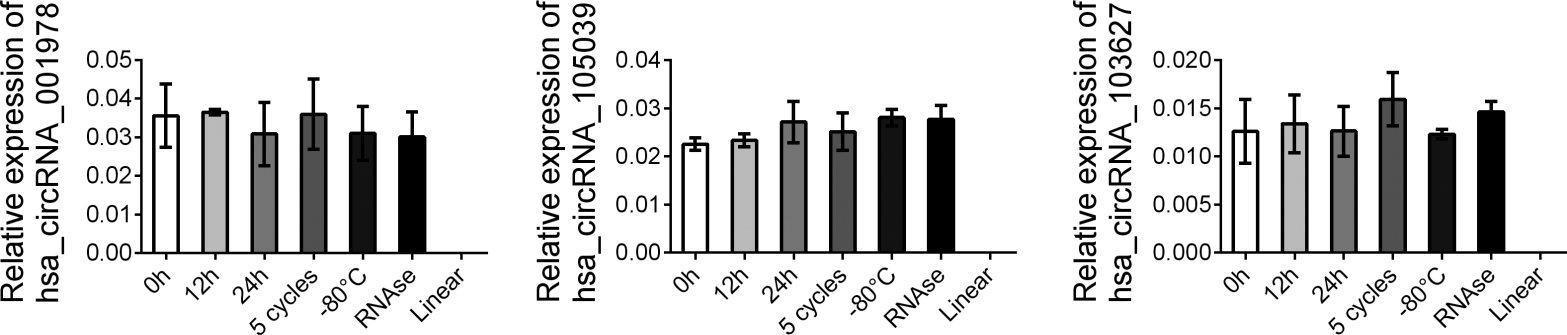
Relative expression of three circRNAs in different condition. The samples were placed at room temperature for 12 and 24 h and subjected to three consecutive freeze‐thaw treatments

To verify the stable presence of the three circRNAs in human plasma, plasma samples from randomly selected healthy patients were treated in different ways to test the stability of circRNA expression in different environments. The samples were placed at room temperature for 12 and 24 h. In addition, the plasma samples were subjected to three consecutive freeze‐thaw treatments. It was found through detection that the expression levels of these three circRNAs in the above plasma did not altered significantly indicating that the three circRNA panel could be stable expressed in human plasma samples (Figure 6).

## 4 DISCUSSION

At present, the diagnosis of CRC mainly relies on colonoscopy, imaging and tumor biomarker detection. However, due to the unsatisfactory results of these methods, patients with CRC are usually diagnosed as intermediate to late stage. In addition, as the gold standard, colonoscopy has not been widely promoted due to the high cost of examination, the painful process of examination, and the poor health awareness of the public.^17^ Similarly, imaging examination is difficult to be used for early diagnosis of colorectal cancer due to its poor identification. In terms of diagnostic markers, carcinoembryonic antigen and CA19‐9 has been widely applied in the diagnosis of tumor, but previous studies show that CEA is more suitable for being used in the detection of for tumor recurrence and metastasis; in addition, the CEA levels in intestinal inflammation and adenoma has increased in the tumor, for early colorectal cancer its poor sensitivity and specific degree, CA19‐9 is a good predictor of tumor metastasis.^18^ Therefore, finding new tumor markers with high sensitivity and specificity is very important for the diagnosis of CRC.

It is worth noting that in the current research, more and more researchers have found that circRNAs play an important role in many human diseases. In addition, many circRNAs can be expressed in plasma. For example, circ_0124554 blocked the ubiquitination of AKT promoting the skip lymphovascular invasion on hepatic metastasis in colorectal cancer.^19^ Due to the circRNA specific circular structure, they can be stably expressed in human body fluids, including plasma and urine, and thus have the ability to serve as biomarkers.^20^ It has been shown that circRNA can be used as an important marker of early tumor occurrence and predict the occurrence of gastric cancer in gastrointestinal tumors.^21^ In colorectal cancer, many studies have found that circRNAs can predict the occurrence and development of colorectal cancer. In addition, some specific circRNAs have good predictive effects on liver metastasis of colorectal cancer ^22^, ^23^. However, previous studies on circRNAs as a diagnostic marker all took colorectal cancer patients and healthy people as the research objects, lacking the case process of the transformation from precancerous lesions, such as adenoma, to colorectal cancer, and the false‐positive phenomenon of the marker in benign tumors could not be identified. In this study, as a marker for the early diagnosis of colorectal cancer, benign tumors were included as a reference in addition to the routine focus on the abnormal increase of circRNAs in colorectal cancer patients compared with healthy people. In addition, the application of risk stratification analysis can avoid low sensitivity and low specificity markers in the screening process and provide a guarantee for the reliability.

After the above screening, it was found in this study that hsa_circ_001978, hsa_circ_105039, and hsa_circ_103627 could better identify colorectal cancer patients from intestinal adenoma patients and healthy people and could be stable and highly expressed in the plasma of colorectal cancer patients. At present, the mechanism of these three circRNAs in colorectal cancer has not been reported. Only studies have shown that circRNA_105039 plays a spongy role in promoting CCND2 expression and thereby promoting cardiomyocyte differentiation through the absorption of miR‐17. Therefore, the next step of this study is to further explore whether these three circRNAs can promote the occurrence and development of colorectal cancer tumors and their specific mechanisms. In addition, as a standard diagnostic marker, we need a large sample of independent centers to revalidate the sensitivity and specificity of the marker. It is particularly important to detect whether these three circRNAs are highly expressed in plasma of other gastrointestinal cancer patients to exclude the occurrence of false positive.

## 5 CONCLUSION

Overall, we discovered a plasma circRNA panel among abundant samples that distinguishes CRC from healthy and CRA. The results showed that this plasma circRNA panel has a significant clinical value in the early diagnosis of CRC, so more patients can avoid missing the treatment window phase and accept more effective and thorough therapy to improve long‐term survival.

## CONFLICT OF INTEREST

No potential conflict of interest was reported by the authors.

## AUTHOR CONTRIBUTIONS

XC conceived and designed the experiments; LQ and YP performed the experiments and contributed to reagents/materials/analysis tools; LQ AND MT analyzed the data; All authors contributed to the article and approved the submitted version.

## Data Availability

The datasets used and/or analyzed during the current study are available from the corresponding author on reasonable request.
